# Thrombelastographic haemostatic status and antiplatelet therapy after coronary artery bypass surgery (TEG-CABG trial): assessing and monitoring the antithrombotic effect of clopidogrel and aspirin versus aspirin alone in hypercoagulable patients: study protocol for a randomized controlled trial

**DOI:** 10.1186/1745-6215-13-48

**Published:** 2012-04-27

**Authors:** Sulman Rafiq, Pär Ingemar Johansson, Mette Zacho, Trine Stissing, Klaus Kofoed, Nikolaj Bang Lilleør, Daniel Andreas Steinbrüchel

**Affiliations:** 1 Department of Cardiothoracic Surgery, The Heart Centre, Rigshospitalet, Copenhagen University Hospital, Copenhagen, Denmark; 2 Capital Region Blood Bank, Section for Transfusion Medicine, Rigshospitalet, Copenhagen University Hospital, Copenhagen, Denmark; 3 Department of Radiology, Rigshospitalet, Copenhagen University Hospital, Copenhagen, Denmark; 4 Department of Cardiology, The Heart Centre, Rigshospitalet, Copenhagen University Hospital, Copenhagen, Denmark

**Keywords:** Coronary artery bypass, CABG, Thrombelastography, TEG, Multiplate aggregometry, Graft patency, Thromboembolic, Antiplatelet, Clopidogrel, Aspirin

## Abstract

**Background:**

Hypercoagulability, assessed by the thrombelastography (TEG) assay, has in several observational studies been associated with an increased risk of post-procedural thromboembolic complications. We hypothesize that intensified antiplatelet therapy with clopidogrel and aspirin, as compared to aspirin alone, will improve saphenous vein graft patency in preoperatively TEG-Hypercoagulable coronary artery bypass surgery (CABG) patients and reduce their risk for thromboembolic complications and death postoperatively.

**Methods/Design:**

This is a prospective randomized clinical trial, with an open-label design with blinded evaluation of graft patency. TEG-Hypercoagulability is defined as a TEG maximum amplitude above 69 mm. Two hundred and fifty TEG-Hypercoagulable patients will be randomized to either an interventional group receiving clopidogrel 75 mg daily for three months (after initial oral bolus of 300 mg) together with aspirin 75 mg or a control group receiving aspirin 75 mg daily alone. Monitoring of antiplatelet efficacy and on-treatment platelet reactivity to clopidogrel and aspirin will be conducted with Multiplate aggregometry. Graft patency will be assessed with Multislice computed tomography (MSCT) at three months after surgery.

**Conclusions:**

The present trial is the first randomized clinical trial to evaluate whether TEG-Hypercoagulable CABG patients will benefit from intensified antiplatelet therapy after surgery. Monitoring of platelet inhibition from instituted antithrombotic therapy will elucidate platelet resistance patterns after CABG surgery. The results could be helpful in redefining how clinicians can evaluate patients preoperatively for their postoperative thromboembolic risk and tailor individualized postoperative antiplatelet therapy.

**Trial registration:**

Clinicaltrials.gov Identifier NCT01046942

## Background

Coronary artery bypass surgery (CABG) is conducted in approximately one million patients a year worldwide. The success of the operation is highly dependent on the vascular bypass remaining open. Saphenous vein graft (SVG) is the most commonly used bypass conduit [1]. SVG occlusion is reported from 5% to 15% within the first year [1,2]. Thrombosis of the SVG is the primary reason for occlusion in the first days and weeks after surgery; hereafter the processes of intimal hyperplasi and progressive graft atheroschlerosis are assumed to gradually play a greater role in graft occlusion [3,4].

### Hypercoagulability and thromboembolic risk

Recent observational studies have shown an association between hypercoagulability measured by thrombelastography (TEG) defined by increased thrombin-induced platelet-fibrin clot strength (TEG-Hypercoagulable) and postoperative/post-interventional thromboembolic complications. Gurbel and colleagues reported that in patients undergoing percutaneous coronary intervention (PCI), 60% of TEG-Hypercoagulable patients developed an ischemic event post-intervention, while this only was the case in 9% of TEG-Normocoagulable patients, *P* <0.0001 [5]. Another observational study in patients undergoing major non-cardiac surgery found that 8 out of 95 (8.4%) of TEG-Hypercoagulable patients had a postoperative thromboembolic complication, while only 2 out of 145 (1.4%) TEG-Normocoagulable patients experienced thromboembolic episodes, *P* = 0.016 [6]. Also, in critically ill patients in the surgical intensive care unit, TEG-Hypercoagulable status has been demonstrated to yield a significantly greater risk for thromboembolic complications [7]. Our group has conducted a prospective observational study of 200 consecutive CABG patients, and found that TEG-hypercoagulability (defined as TEG maximum amplitude (MA) above 69 mm) was prevalent in 87 patients (43.5%) before surgery, and preoperative TEG-Hypercoagulable patients had a higher risk of a combined endpoint of myocardial infarction (MI), stroke and death after 30 days, as compared to TEG-Normocoagulable patients, 17.2% vs. 6.6%, *P* = 0.019 [8].

Based on the above findings it could be speculated whether TEG-Hypercoagulable patients may benefit from intensified antiplatelet therapy to reduce thromboembolic risk after surgery/intervention.

Furthermore, it has been demonstrated that surgery itself induces hypercoagulability, both after CABG and major non-cardiac surgery [6,9]. Our study will help elucidate if the extent of this increase in patients who are already TEG-hypercoagulable preoperatively, is an independent risk factor.

### Antiplatelet therapy after CABG

Antiplatelet therapy with aspirin is well established after CABG. Graft occlusion rates in the 1980s were reduced from 20 to 30% in the first year after surgery to 10 to 15%, if aspirin therapy was restarted 6 to 24 hours after surgery [3]. Clopidogrel is a thienopyridine antiplatelet agent that inhibits adenosine diphosphate (ADP)-receptors on the platelet surface (P2Y12 receptors), thereby reducing platelet activation and aggregation [10]. Dual antiplatelet therapy with clopidogrel in addition to aspirin is well established in the treatment of acute coronary syndromes and in PCI patients, and has been shown to significantly reduce cardiovascular events and death as compared to aspirin treatment alone in these patients [10-12]. However, these studies have also reported that dual antiplatelet therapy significantly increases bleeding risk [10-12].

The role of dual antiplatelet therapy after CABG is still not clear. Current guidelines from the European Society of Cardiology (ECS) and European Association of CardioThoracic Surgeons (EACTS) recommend dual antiplatelet therapy in acute coronary syndrome patients after CABG [13]. These recommendations are primarily based on the “Clopidogrel in Unstable angina to prevent Recurrent ischemic Events” (CURE) and “Clopidogrel Versus Aspirin in Patients at Risk of Ischemic Events” (CAPRIE) trials [12,14]. It should be noted though, that the subgroup study of the CURE trial failed to demonstrate any beneficial effect of clopidogrel after CABG [14]. Furthermore, the CAPRIE investigators performed a subgroup analysis of patients who have had prior cardiac surgery, but did not stratify for different types of procedures (valve surgery, CABG and so on) and also did not take time from surgery to inclusion in the study (up to years) into consideration [12]. Consequently, these data do not support the routine use of clopidogrel in addition to aspirin after CABG in patients with acute coronary syndrome undergoing surgical revascularization, and this has been corroborated by a recent Cochrane review [15].

No randomized trial to date has examined the role of clopidogrel after cardiac surgery in acute coronary syndrome patients. The randomized trials have focused on the CABG patient population as a whole. Last year the long awaited “Clopidogrel After Surgery for Coronary Artery Disease” (CASCADE) trial was published. The primary endpoint focused on potentially reduced SVG intimal hyperplasi in patients on dual antiplatelet therapy (clopidogrel and aspirin) as compared to aspirin and placebo therapy. As a secondary endpoint, SVG patency was also examined. The study did not demonstrate significant differences among antiplatelet regimens [16]. “The Preoperative Aspirin and Postoperative Antiplatelets in Coronary Artery Bypass Grafting” (PAPA-CABG) trialists have published pilot data from the first 100 patients randomized to clopidogrel and aspirin vs. aspirin alone, reporting no difference in SVG patency at 30 days measured by cardiac computed tomography [17]. Gurbuz and associates have published data from 591 consecutive off-pump coronary artery bypass surgery procedures performed by one surgeon. There was no randomization but historical controls. They demonstrated a significant positive clinical effect of dual antiplatelet therapy on the endpoints of postoperative MI and death [18]. Gao and colleagues published data from a randomized clinical trial in 2010 showing that SVG patency assessed by multi-slice computed tomography (MSCT) three months after CABG showed significant improvement in SVG patency in patients receiving dual antiplatelet therapy with clopidogrel and aspirin vs. aspirin alone, 92% vs. 86%, *P* = 0.043 [19].

### High on-treatment platelet reactivity and thromboembolic risk

In recent years, there has been a growing acknowledgement of the association between insufficient platelet inhibition from instituted antiplatelet therapy (that is, clopidogrel and/or aspirin) and thromboembolic complications. Studies in PCI patients have demonstrated a significantly higher risk for post-procedure thromboembolic episodes in individuals that demonstrate high on-treatment platelet reactivity to ADP [3,20-22]. Few studies have demonstrated that after conventional CABG with cardio-pulmonary bypass up to 50% of the patients will be transiently aspirin resistant, and this high on-treatment platelet reactivity has been correlated to early SVG thrombosis after CABG [3,23]. The platelet reactivity patterns of clopidogrel after cardiopulmonary bypass have not been examined. We will monitor platelet reactivity in our trial to elucidate the patterns and degree of high on-treatment platelet reactivity of aspirin and clopidogrel in the immediate postoperative period and three months after CABG.

### Hypothesis

We have asked the following research questions: Can intensified antiplatelet therapy with the addition of clopidogrel to aspirin as compared to routine aspirin monotherapy significantly improve graft patency at three months in TEG-Hypercoagulable patients? Can dual antiplatelet therapy significantly reduce the heightened risk for thromboembolic complications and cardiovascular death in TEG-Hypercoagulable patients? Does failure of instituted antiplatelet therapy to inhibit platelet aggregation increase patients risk for SVG occlusion and thromboembolic events after CABG?

## Methods/Design

### Study population

Patients over the age of 18 referred to our tertiary institution (Department of Cardio-thoracic Surgery, Rigshospitalet, Copenhagen University Hospital, Denmark) for isolated non-emergent CABG procedure were screened for eligibility. See inclusion/exclusion criteria in Table 1. Patients are enrolled from November 2008 to March 2013.

**Table 1:** Inclusion/exclusion criteria



**Inclusion Criteria**
· Elective/subacute multivessel CABG
· Isolated CABG procedure, no concomittant surgery
· Age >18 years
· Able to give informed consent
**Exclusion Criteria**
· Myocardial infarction >48 h of surgery
· Prior CABG surgery within one month
· Cardiac Shock within 48 h of surgery
· Atrial fibrillation
· Anticoagulation therapy with VKA
· ICH/TCI within 30 days
· Prior peptic ulcer
· Platelet count >150 E9
· Ongoing bleeding
· Known platelet disease
· Allergic to aspirin or clopidogrel
· Liver disease with elevated ALAT/ASAT >1.5x normal
· Creatinine >0.120 mmol/l
· Contrast allergy
· Alcohol or narcotics abuse
· Pregnancy
· Geographically not available for follow-up

This table shows the inclusion and exclusion criteria in the TEG-CABG trial

### Design and randomization procedure

**Figure 1:**
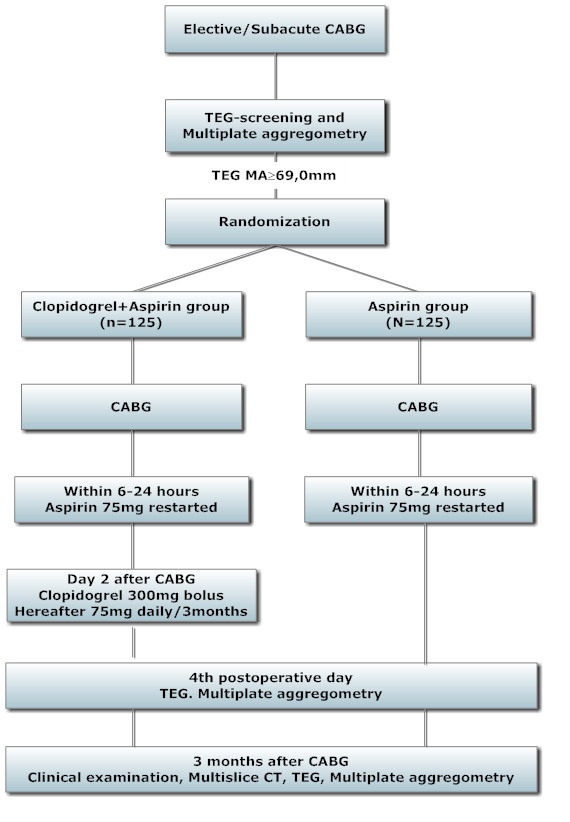
Study flow chart.

The TEG-CABG trial is a prospective open label, randomized clinical trial with blinded evaluation of the primary endpoint (graft patency evaluation by 320 slice-MSCT). Figure 1 shows a flowchart of the study.

The study nurse, utilizing the sealed envelope technique, randomizes 250 TEG-Hypercoagulable (TEG MA >69 mm) patients on the day before CABG, allocating 125 patients to the interventional group (receiving clopidogrel for three months and lifelong aspirin) and 125 TEG-Hypercoagulable patients to a control group (receiving lifelong aspirin only).

TEG, Multiplate aggregometry and routine blood samples are obtained the day before surgery, on Day 4 after CABG, and at three months postoperatively.

MSCT is conducted at three months after surgery.

### Surgical procedures

All CABG procedures are performed through median sternotomy. At our institution approximately 97% of CABG procedures are performed on-pump with cardiopulmonary bypass and extracorporal circulation (ECC). Patients on ECC are heparinised to maintain an acquired coagulation time (ACT) above 480s, and this is reversed with protaminesulphate (1:1) after finalization of ECC. All patients on ECC receive a total of four grams tranexamic acid during surgery.

The left internal mammarian artery (LIMA) is the preferred conduit for anastomoses to the left anterior descending artery (LAD), for all other anastomoses SVG conduits are preferred.

There is no specific team assigned or any restrictions of cardiac surgeons and/or anesthetists to perform procedures on patients in this trial.

### Perioperative anticoagulation

Routinely, aspirin and clopidogrel are discontinued five days before surgery. In patients with acute coronary syndrome 40 mg of Clexane (enoxaparin- a low-molecular-weight heparine (LMWH)) twice daily or 2.5 mg of Arixtra (Fondaparinux) once daily is administered subcutaneously until the day before surgery. After surgery, only off-pump patients received Clexane 40 mg s.c. twice daily until discharged from hospital, all other patients do not receive LMWH after CABG.

### Description of medical intervention

Aspirin 75 mg is restarted within 24 hours of surgery, in accordance with the current recommendations from ECS/EACTS [13]. In the intervention group clopidogrel is started at Day 2 after surgery, with a 300 mg oral bolus to achieve greater initial platelet inhibition [24,25]. Thereafter, clopidogrel therapy is continued with a daily dose of 75 mg for 90 days. Our study is the first study in CABG patients to utilize initial oral bolus of 300 mg clopidogrel. Greater loading doses of clopidogrel (that is, 600 mg) were not utilized due to bleeding concerns - in particular GI bleeding - in the postoperative setting, expressed by the cardiac surgeons at our institution.

Patients will receive study medication (clopidogrel) from the study nurse for the whole study period. At three months the patients are requested to bring in their remaining medication, so compliance can be assessed.

### Outcome measures

Primary:

Graft patency assessed at three months by MSCT will be significantly improved in TEG-Hypercoagulable patients allocated to dual antiplatelet therapy with clopidogrel and aspirin as compared to aspirin alone.

Secondary:

1. TEG-Hypercoagulable patients will suffer fewer thromboembolic events (that is, MI, stroke, pulmonary embolism, gastrointestinal ischemia, deep vein thrombosis) or cardiovascular death when on clopidogrel and aspirin vs. aspirin alone.

2. Assess if the degree of surgically induced hypercoagulability itself is an independent factor of graft occlusion, thromboembolic events and death.

3. High on-treatment platelet reactivity in spite of Aspirin and/or clopidogrel treatment, as evaluated by Multiplate aggregometry, increases the risk of thromboembolic events after CABG surgery.

All outcome measures will be evaluated at three months postoperatively.

### Thrombelastography (TEG)

Until recently, detection of platelet hyper-reactivity demanded sophisticated and time consuming assays. With the modernization and computerization of the TEG methodology, it has gained more clinical interest in specialities beyond cardiac and liver surgery, in which TEG analysis has been used to guide transfusion regimens since the 1980s [26]. TEG measures kinetics of clot formation, clot strength, platelet function and fibrinolysis, and thus provides a global picture of the patient’s hemostatic ability and the viscoelastic properties of the clot [26,27]. The analysis is performed on whole blood, in contrast to routine plasma-based coagulation tests [26]. For TEG-analysis, whole blood is collected in a 3.2% trisodium citrate vacutainer (Greiner Bio-One, Kremsmünster, Austria) and analyzed within 90 minutes after collection by use of a computerized TEG coagulation analyser (TEG model 5000, Haemoscope Corporation, Niles, IL, USA), but not earlier than 30 minutes after collection.

**Figure 2:**
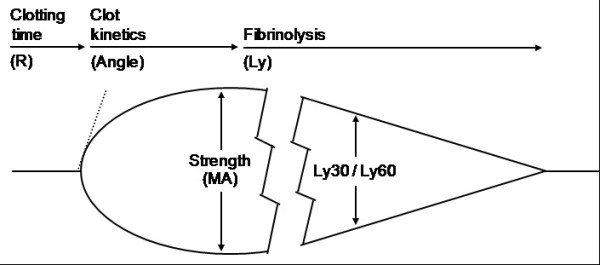
TEG tracing.

In the TEG analyser, whole blood is incubated in a heated cup (37°C). Within the cup a torsion wire, which can monitor movement, is suspended. In our series we utilize kaolin as a potent initiator of coagulation other activators of coagulation can be used [23]. The cup starts rotation, and as fibrin forms between the cup and the pin, the rotation is transmitted from the cup to the pin, and the movement of the pin is registered by a computer and the TEG trace is generated [26,27]. The TEG trace graphically depicts different stages of the hemostatic process; clotting time, kinetics, strength and lysis (*See Figure 2) [26,27]. The TEG maximal amplitude (MA) depicts the maximal strength of the clot, and MA values above or equal to 69 mm are diagnosed TEG-Hypercoagulable, according to the manufacturer.

### Multiplate aggregometry

Because of the generation of thrombin secondary to the kaolin (a very potent initiator of coagulation) activation in the TEG assay, the effects of antithrombotic agents such as aspirin (acetylsalicylicacid), non-steroid anti-inflammatory drugs (NSAID), and ADP-receptor inhibitors, such as clopidogrel, cannot be evaluated. Therefore, the effects of aspirin and clopidogrel on platelet inhibition are evaluated by Multiplate impedance aggregometry (Multiplate, Dynabyte Medical, Munich, Germany). The assay consists of two silver-coated copper electrodes immersed in a whole blood sample [28]. The assay is based on the principle that platelets are non-thrombogenic in a resting state. Upon admission of specific receptor agonists, the platelets will begin to adhere to the electrodes, if the receptors are not blocked by medication. As the platelets adhere to the electrodes, the resistance between the electrodes will increase and the impedance is registered by a computer [28]. If addition of ADP to a clopidogrel-treated patient sample does not trigger any significant adherence of platelets to the electrodes, this means the drug is successfully blocking the ADP-receptors. If addition of ADP does trigger platelet adhesion, the patient’s ADP receptors are not successfully blocked by clopidogrel. The results of this assay are shown to correlate with the development of thrombotic complications in PCI patients [21]. This assay has been validated against light transmission aggregometry, which is the “gold standard” in platelet aggregation assays [28].

In the present study, we use the following platelet agonists: ADP (testing for clopidogrel effect), ASPI (arachnoid acid, testing for aspirin effect) and TRAP (thrombin receptor-activating peptide, testing for maximum response of platelets) [28].

### Multislice computed tomography (MSCT)

MSCT technology is rapidly evolving in cardiac imaging. MSCT allows for assessment of cardiac structures with high spatial and temporal resolution. MSCT with 16-slice and 64-slice scanners has been validated for assessing graft patency after CABG [29-31]. Radiation exposure with the evolvement of scanners and scanner software is brought to a minimum, and is now comparable with the radiation exposure of a standard cardiac angiography [30]. The first 25 patients were scanned with a 64-slice scanner (Toshiba Aquilion 64, Tokyo, Japan); due to an upgrade of the scanner, the rest of the patients included are scanned using a 320-slice scanner (Toshiba Aquilion ONE, Tokyo, Japan). The scanning protocol is as follows: gantry rotation time 350 ms, detector collimation 0.5 x 320. Tube voltage and current are chosen based on the patient’s body mass index ranging between 100 and 120 kV, and mA between 280 and 500. An intravenous contrast media (Visipaque 320 mg I/ml, GE Healthcare, Buckinghamshire, UK) is infused using a flow rate of 6 ml/s followed by a saline chaser. The contrast dye volume used is individually calculated according to patient body mass index (100 to 130 ml). Image interpretation is performed using commercially available software (Vitrea, version 3.0.1, Vital Images, Minnetonka, Minnesota, USA). Grafts are evaluated by two experts in cardiac MSCT these are blinded to patients coagulation and randomization status in the trial.

### Sample size

All patients receive a pedicled left internal thoracic artery graft at our institution; therefore, every patient on average will receive an additional two SVGs. Graft patency for SVGs is approximately 90% after one year. Under the assumption that TEG-Hypercoagulable patients have a lower SVG patency of 80%, and that dual antiplatelet therapy will raise the graft patency of these patients to the rate in uncomplicated patients (90%), calculations based on the Fischer exact test with 80% power and 5% significance level, show the following:

#### ***Improvement of graft patency from 80% to 90%: 219 grafts per group***

With an average of 2 SVGs per patient; 219/2 = 110 patients in each group.

We will include 125 patients in each group to account for “drop-outs” and be able to detect at a significance level of 5% or better.

### Data collection

Preoperatively, during surgery, at Days 3 to 5 after surgery, at discharge and then again three months after surgery, data are collected and entered in the case report form (CRF). The following data will be collected: demographic data, past medical history with special emphasis on cardiac and thromboembolic disease and medication. Intraoperatively data will also be included, this includes a description of the quality of SVGs. In-hospital events, such as MI, stroke and bleeding complications, will be registered. Throughout the study period, blood component transfusion requirements will be registered. The data are transferred from the CRF to a secure database by the project coordinator. The local data safety set-up has been approved of the Danish Data Protection Agency.

### Data and statistical analysis

Results will be included in the final analysis from patients who have complied with correct intake of study medicine. Patients, who fail to do so, will be excluded. Patients, who die during the study period, will be included in a survival analysis. Patients who must discontinue study medication due to a serious adverse event (SAE) will be followed closely until their condition is resolved and relevant treatment is completed. SAEs will be reported to the local health authorities.

Interim analysis will be conducted when 100 TEG-hypercoagulable patients have been enrolled and undergone three-month MSCT.

For categorical variables, χ^2^ tests or Fisher’s exact test will be used as appropriate. For continuous variables, analysis will be performed with the two-sample t-test, after ensuring normal distribution of the data. Data with no normal distribution will be analyzed with the Wilcoxon’s rank-sum test. *P*-values below 0.05 will be statistically significant. Multivariate analyses will be performed, including both demographic and procedural confounding factors. Survival analysis will include a Kaplan-Meyer plot.

### Ethics and trial registration

The study is conducted in accordance with the Helsinki 2 declaration and has been approved by the Danish Research Ethics Committee in March 2008 (H-C-2007-0057). The study is registered at EUDRACT (European Union Drug Regulating Authorities Clinical Trials) and at clinicaltrials.gov (Identifier NCT01046942).

## Discussion

The evidence for clopidogrel in addition to aspirin after CABG is divergent, although most studies have not been able to demonstrate any beneficial effect of dual antiplatelet therapy. Meanwhile, emerging evidence from observational studies has demonstrated a significantly higher thromboembolic risk in both PCI and CABG patients that are TEG-Hypercoagulable. The TEG-CABG trial is the first randomized trial to investigate preoperatively TEG-Hypercoagulable CABG patients. The rationale being that this is a patient population with a heightened thromboembolic risk (including graft occlusion) due to the hypercoagulability, but, hence, also is at low risk for bleeding on dual antiplatelet therapy. We hypothesize that this subgroup of CABG patients will benefit the most on a dual antiplatelet regimen both in regards to SVG patency and thromboembolic complications in general.

Examining hemostatic ability and status before and after surgery/intervention with TEG, we believe will be the future for postoperative thromboembolic risk evaluation. Including Multiplate aggregometry measurements in our trial will also help elucidate if resistance measurements after cardiac surgery can help better tailor antiplatelet regimens. These points become even more important as the patients referred to cardiac surgery get older and have more co-morbidities, and, hence, have limited physiological reserves, where graft occlusions and even small thromboembolic episodes can have detrimental effects on overall health and survival.

### Limitations

Due to lack of funding no placebo drug is used, and this is why the open label design was chosen. However, we find it plausible to assume that the primary endpoint of graft patency after three months will not be affected by the lack of placebo drug in the control group, as the MSCT evaluators are blinded.

## Conclusions

The TEG-CABG trial is the first randomized trial to investigate whether TEG-Hypercoagulability, before and after CABG, is predictive of trombembolic events and death, and if intensified antiplatelet therapy can reduce this risk. The primary end-point will be SVG patency at three months postoperatively. The results of this study may help redefine how patients undergoing coronary surgery could be evaluated individually in the future for their postoperative thromboembolic risk, and enable clinicians to tailor antiplatelet therapy to counter this risk.

## Trial status

Currently 135 patients are randomized in this trial, and inclusion is ongoing.

## Abbreviations

ADP: Adenosine diphosphate; CABG: Coronary artery bypass grafting surgery; CAPRIE: Clopidogrel versus aspirin in patients at risk of ischemic events trial; CASCADE: Clopidogrel after surgery for coronary artery disease trial; CRF: Case report form; CURE: Clopidogrel in Unstable angina to prevent recurrent ischemic events trial; EUDRACT: European Union Drug Regulating Authorities Clinical Trials; LAD: Anterior descending artery; LIMA: Left internal mammarian artery; LMWH: Low-molecular-weight heparine; MA: Maximal amplitude; MSCT: Multislice computed tomography; NSAIDs: Non-steroid anti-inflammatory drugs; PAPA-CABG: The Preoperative aspirin and postoperative antiplatelets in coronary artery bypass grafting; PCI: Percutaneous coronary intervention; SAE: Serious adverse event; SVG: Saphenous vein graft; TEG: Thrombelastography.

## Competing interests

The authors declare that they have no conflict of interest.

## Authors’ contributions

All authors made a substantial contribution to this manuscript and study design in regards to conception, design and drafting. All authors have read and approved the final manuscript.
